# Ovarian Carcinoma Uncloaked by Hydatidosis: An Intraoperative Epiphany

**DOI:** 10.7759/cureus.11276

**Published:** 2020-10-31

**Authors:** Talal Almas, Tarek Khedro, Salman Hussain, Reema Alsufyani, Muhammad Kashif Khan

**Affiliations:** 1 Internal Medicine, Royal College of Surgeons in Ireland, Dublin, IRL; 2 Surgical Oncology, Federal Government Poly Clinic (Post Graduate Medical Institute), Islamabad, PAK; 3 Surgical Oncology, Maroof International Hospital, Islamabad, PAK

**Keywords:** ovarian cyst, hydatidosis, ovarian hydatidosis

## Abstract

Hydatidosis is an infective ailment that is caused by the parasite echinococcus granulosus. The parasitic infection typically produces cysts filled with excessive quantity of fluid and most commonly afflicts the liver. While secondary hydatidosis remains exceedingly rare, cases of hydatidosis in organs such as the ovaries and the fallopian tubes have been documented in the literature. In such instances, the patients present with a vague constellation of symptoms, including abdominal distension and vague abdominal pain. Herein, we elucidate the case of a female patient who presented with massive abdominal swelling on a background history significant for hydatid liver disease. Intraoperative findings included a left adnexal mass, which was eventually established to be an ovarian adenocarcinoma coexisting with secondary ovarian hydatidosis.

## Introduction

Echinococcus granulosus is often regarded as the culprit etiology underlying hydatid cysts. Hydatid cysts are fluid-filled cysts that can grow to exorbitant proportions before they are detected. While the infection, also termed hydatidosis, is prevalent in South America, the Mediterranean area, and Eastern Europe, its endemicity and persistence in southeast Asia have not previously been documented [1]. The cysts of hydatidosis are noted to infiltrate the liver in roughly 70% of the cases [2]. Contrarily, involvement of other organs, including the kidneys and the ovaries, is very rare [2]. Notably, the female reproductive system has been implicated in cases of hydatidosis in merely 0.5% of all reported cases [2,3]. While these cysts can affect any part of the female reproductive system, the majority afflict the ovaries. When it does occur, ovarian hydatidosis presents with a constellation of symptoms, including pelvic pain and dysmenorrhea [3]. Due to its vague clinical manifestations, ovarian carcinoma can often masquerade as ovarian hydatidosis, obscuring a timely diagnosis [4]. In this study, we present an extremely rare case of unilateral secondary ovarian hydatidosis. A surgery to excise the ovarian cyst was performed and revealed the coexistence of ovarian carcinoma. 

## Case presentation

**Figure 1:**
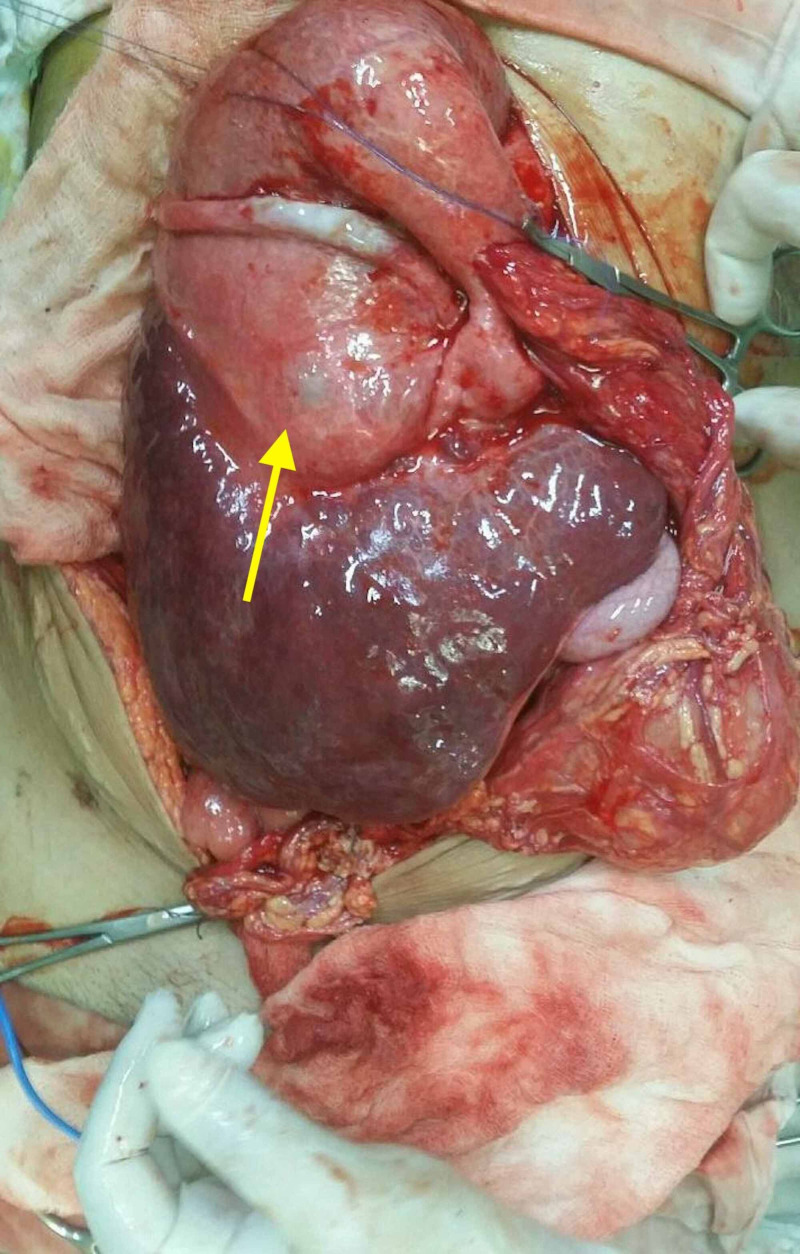
Intraoperative image showing a simple liver cyst (yellow arrow) replacing segments five and eight of the liver.

We chronicle the case of a 60-year-old female who presented with massive ascites and vague, generalized abdominal discomfort. Clinical examination revealed diffusely distended abdomen with no rigidity, tenderness or guarding, and obvious hepatosplenomegaly. Pertinently, the patient had first presented to her home clinic six months ago with massive abdominal swelling. Further investigation at this stage had revealed the presence of a hydatid cyst in the patient’s liver. In order to treat the parasitic etiology, the patient was commenced on Albendazole (400 milligrams) after which her clinical symptoms had abated. After completing the therapeutic regimen, the patient was operated five weeks prior to presentation to us. After the surgery, however, the patient developed massive ascites and vague abdominal discomfort, and thus presented to our clinic with her vague constellation of symptoms. Upon presentation, the patient appeared to have an obviously distended abdomen. In order to elucidate the etiology underlying the patient’s current presentation, meticulous diagnostic evaluation was performed. Preoperative radiological investigations divulged a huge simple liver cyst and a unilateral ovarian cyst. Additionally, evaluation of the cancer antigen 125 (CA 125) tumor marker levels was performed but turned out unremarkable. Considering the patient’s prior history of hydatidosis, ovarian hydatidosis was considered as the culprit etiology. Thereafter, a surgical operation using a midline laparotomy was planned. Intraoperatively, an exorbitant hydatid cyst replacing the segments five and eight of the liver was appreciated (Figure 1). 

**Figure 2:**
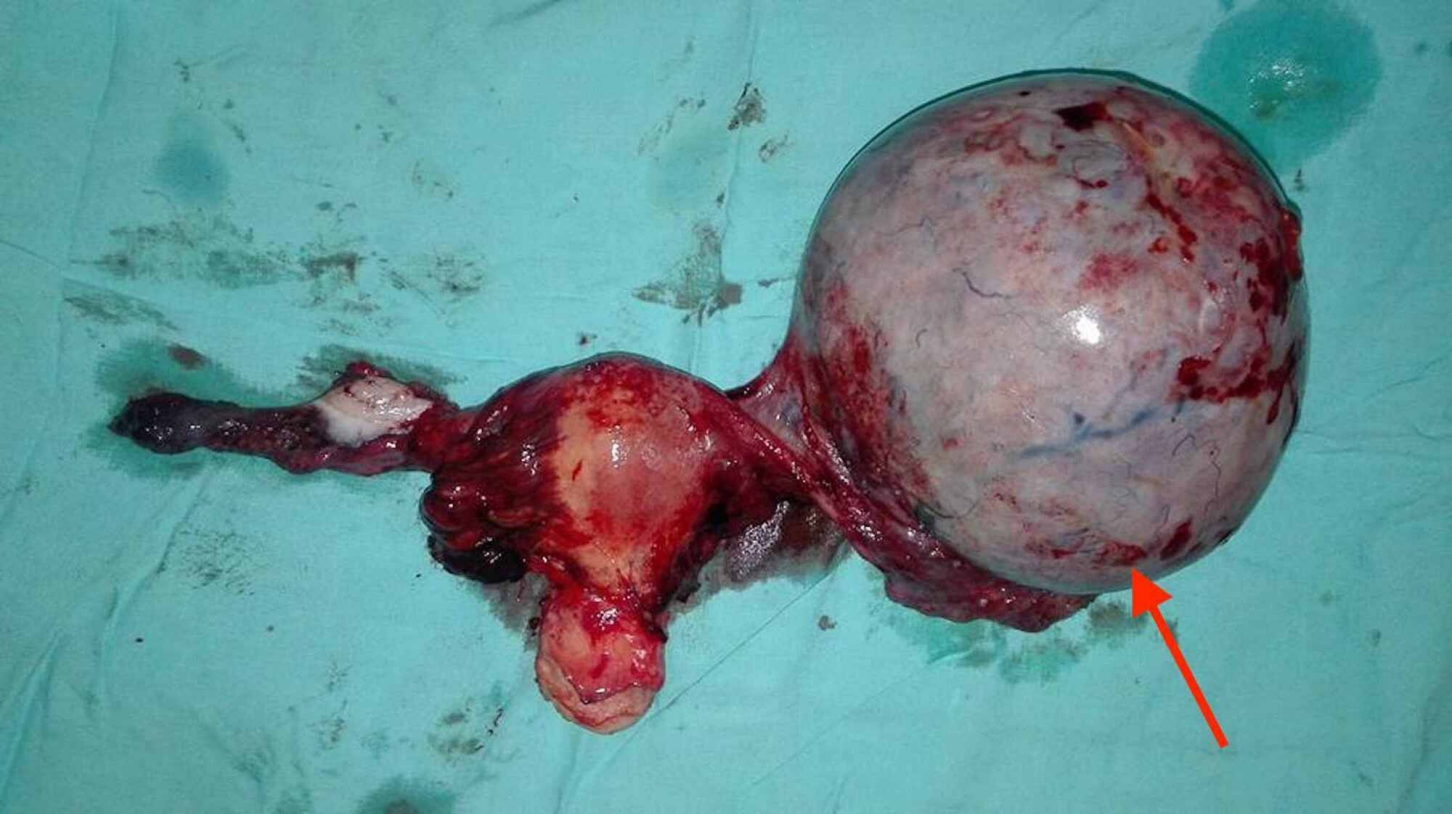
Gross morphology of the specimen obtained from surgery. A normal right ovary with a diseased left ovary (red arrow) can be appreciated.

Furthermore, six liters of ascitic fluid were drained under the cover of intravenous albumin, which explained the patient’s massive abdominal swelling. Interestingly, upon surgery, a left adnexal mass was detected, rousing suspicion of a possible malignancy. Therefore, a bilateral hysterectomy with salpingo-oophorectomy was performed and yielded the sample elucidated in Figure 2.

**Figure 3:**
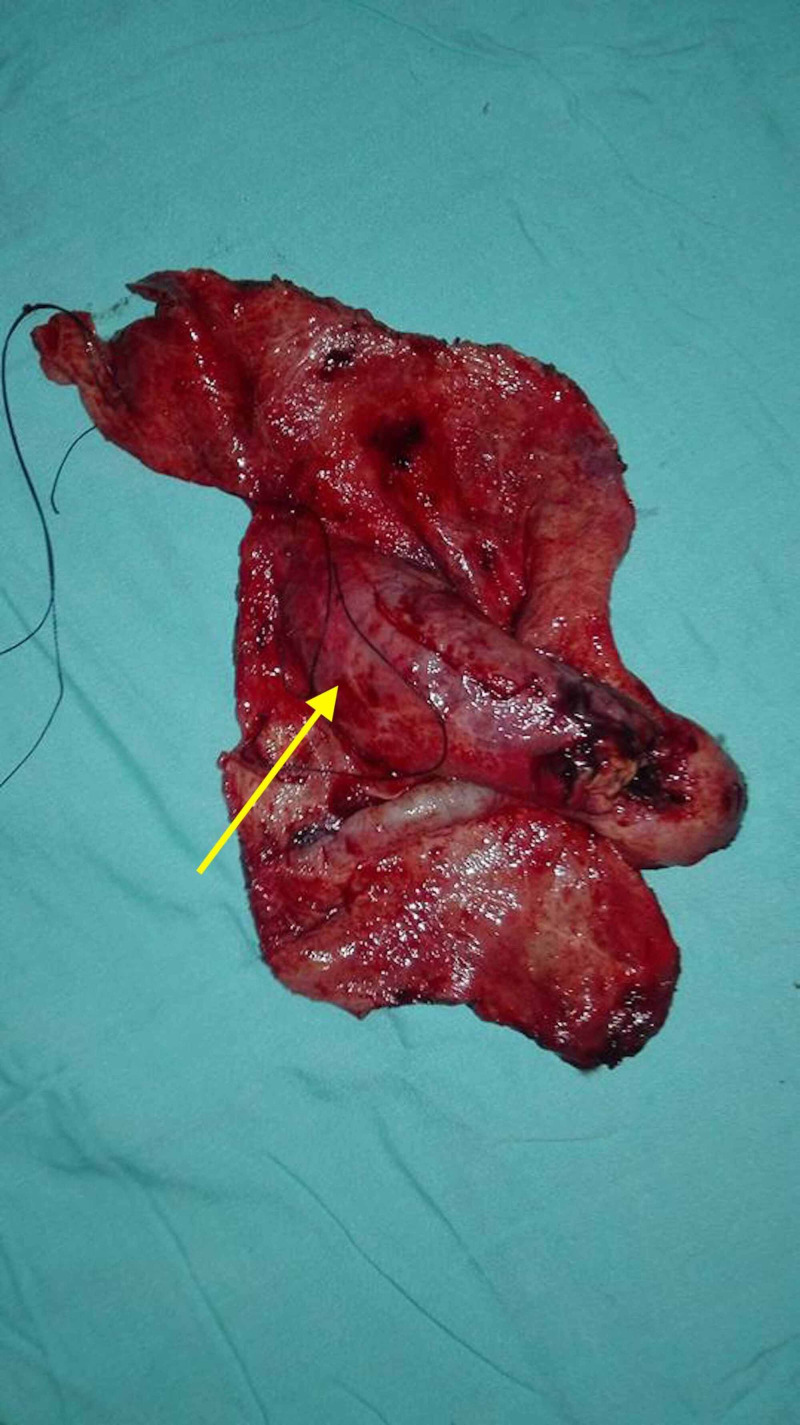
An intraoperative sample delineating the excised cyst wall (yellow arrow).

Additionally, nodules within the pouch of Douglas were excised and sent for histopathological analysis. The analysis revealed the presence of moderately pleomorphic, polyhedral cells with prominent nucleoli. Section through the lesion revealed mucinous epithelium with areas of nuclear stratification, atypia, and occasional mitoses. These findings reaffirmed the presence of a mucinous ovarian adenocarcinoma. A complete de-roofing surgery of the simple cyst along with partial omentectomy was thus performed and yielded the gross specimen in Figure 3.

The patient was discharged in a stable condition postoperatively and continues to do well to date. 

## Discussion

Hydatidosis is a parasitic echinococcal infection that affects the liver in 75% of cases, with most cases being caused by echinococcus granulosus [5]. Cystic echinococcosis has a worldwide annual incidence of 0.2%, with this incidence increased to 2%-6% in endemic populations where sheep and cattle are commonly raised [6]. While hydatidosis can afflict a myriad of organs, including the ovaries, such instances remain exceedingly rare. The pathogenesis of secondary hydatidosis is conjectured to be the result of primary cyst rupture with subsequent dissemination of the parasites [6,7]. To date, only a handful of cases of ovarian hydatidosis have been documented, making ovarian involvement exceedingly rare [6-8]. The clinical presentation of hydatid ovarian cysts often mimics other gynecological malignancies with non-specific clinical manifestations such as abdominal or pelvic pain, nausea, dysmenorrhea, and abdominal distension [6]. This is compounded by the fact that the imaging techniques available, including abdominal ultrasound and computed tomography scanning, are not specific to detecting ovarian hydatid cysts. In one case, sonographic appearance of a secondary hydatid ovarian cyst was described as a well-defined, multicystic mass mimicking a multicystic ovary [8]. It was not until the discovery of a coexistent, morphologically similar multicystic mass in the left lobe of the liver that echinococcosis was suspected. Thus, a prompt diagnosis of more sinister etiologies, such as ovarian carcinoma, can often be obscured, portending adverse disease outcomes. 

Treatment for hydatid disease encompasses conservative surgical approaches such as a partial cystectomy or more radical surgical approaches such as total peri-cystectomy and hysterectomies. In non-operable cases, pharmacological treatment with anti-helminthic medications including praziquantel and benzimidazoles is vital [6]. More importantly, these anti-helminthics are crucial as an adjunctive treatment to surgery. Postoperatively, the recurrence rate of hydatid disease can be up to 25%, and anti-helminthics prescribed preoperatively and postoperatively both significantly reduce this rate [9]. Radical surgical intervention in both hydatid liver and ovarian disease yields better outcomes due to a more definitive cure [10]. In our case, the discovery of a secondary hydatid ovarian cyst, after the initial diagnosis of a sole hydatid liver cyst and its subsequent removal, led to the intraoperative discovery of ovarian carcinoma presenting as a left adnexal mass, thereby necessitating a total abdominal hysterectomy and bilateral saplingo-oopherectomy. Thus, there is an unmet need for more advanced screening measures and diagnostic investigations for hydatid disease that may afford the ability to better distinguish it from the more sinister etiologies.

## Conclusions

Although secondary echinococcosis is rare, physicians should remain cognizant of its possibility in the context of prior hydatid liver disease. In such instances, a detailed clinical examination along with meticulous radiological workup remains imperative. Thus, vague abdominal symptoms should be construed with due caution, keeping in view infective etiologies such as hydatidosis especially in endemic geographical region. 
